# Apoptotic clearance by stem cells: molecular mechanisms for recognition and phagocytosis of dead cells

**DOI:** 10.1038/s41392-024-02091-w

**Published:** 2024-12-30

**Authors:** Feng Liu, Xiaobo Zhou, Ibrahim Akin

**Affiliations:** 1 Department of Cardiology, Angiology, Hemostaseology and Medical Intensive Care, Medical Faculty Mannheim, University Medical Centre Mannheim (UMM), Heidelberg University, 68167 Mannheim, Germany; 2 Department of Cardiovascular Surgery, The Affiliated Hospital, Metabolic Vascular Diseases Key Laboratory of Sichuan Province, Key Laboratory of Cardiovascular Remodeling and Dysfunction, Southwest Medical University, Luzhou, 646000 Sichuan PR China; 3 Key Laboratory of Medical Electrophysiology of Ministry of Education & Medical Electrophysiological Key Laboratory of Sichuan Province, Institute of Cardiovascular Research, Southwest Medical University, Luzhou, 646000 Sichuan PR China; 4 German Center for Cardiovascular Research (DZHK), Partner Site Heidelberg/ Mannheim, Mannheim, Germany

**Keywords:** Cell biology, Stem cells

In a recent article published in *Nature*, Stewart and co-workers provide transformative insights into the mechanisms by which hair follicle stem cells (HFSCs) regulate apoptotic cell clearance to maintain tissue fitness. This study focuses on exploring how HFSCs adopt a temporary phagocytic role at precise moments and balance this duty with their regenerative functions, and identifying key molecules for stem cell sensing and engulfing apoptotic cells.^1^

Apoptotic cell clearance is vital in maintaining tissue integrity and preventing inflammatory and degenerative conditions. Traditionally, professional phagocytes such as macrophages and dendritic cells are responsible for clearing apoptotic cells through a process called efferocytosis.^2^ However, how non-motile, non-professional phagocytes sense and eliminate dying cells while preserving normal tissue function remains unclear. The authors of the *Nature* paper leveraged the unique structure and regenerative properties of the hair follicle to systematically assess HFSCs behavior during the hair cycle. In the resting (telogen) phase, HFSCs reside in a quiescent state, whereas the active (anagen) phase marks their proliferative engagement in hair regeneration. During the catagen phase, the lower portion of the hair follicle undergoes apoptosis, leaving HFSCs in the upper root sheath to clear apoptotic cells. They discovered that HFSCs initiate a highly specific phagocytic response to local apoptotic corpses during the catagen phase. Unlike professional phagocytes,^3^ HFSCs restrict their phagocytic activity to brief, spatially confined periods, aligning closely with the degenerative phases of the hair cycle. To explore mechanisms underlying the recognition and phagocytosis of apoptotic corpses by HFSCs, they unveiled that the phagocytic activity of HFSCs is initiated through a dual-ligand signaling pathway requiring both apoptotic cell-derived lipids, such as lysophosphatidylcholine (LPC), and retinoid signals from neighboring healthy cells. The simultaneous presence of these ligands activates the retinoid X receptor alpha (RXRα) in partnership with retinoic acid receptor gamma (RARγ), which drives the transcriptional upregulation of genes associated with phagocytosis and apoptotic clearance. Concretely, the RXRα–RARγ axis functions as the core regulatory pathway for initiating phagocytic activity in HFSCs in such way that RXRα–RARγ binds to chromatin and opens specific enhancer regions related to apoptotic clearance (Fig. 1). This signaling pathway facilitates the transient upregulation of phagocytic receptors and lysosomal components necessary for corpse digestion, distinguishing HFSCs phagocytosis as an adaptively controlled mechanism rather than a sustained or primary function. The involvement of both apoptotic-derived lipids and healthy cell-produced retinoids implies a dual surveillance system that finely tunes the phagocytic response to local tissue needs without compromising HFSCs’ regenerative roles. Besides, HFSCs have been shown to be able to maintain tissue fitness through accumulating diverse epigenetic memories of distinct experiences.

**Fig. 1:**
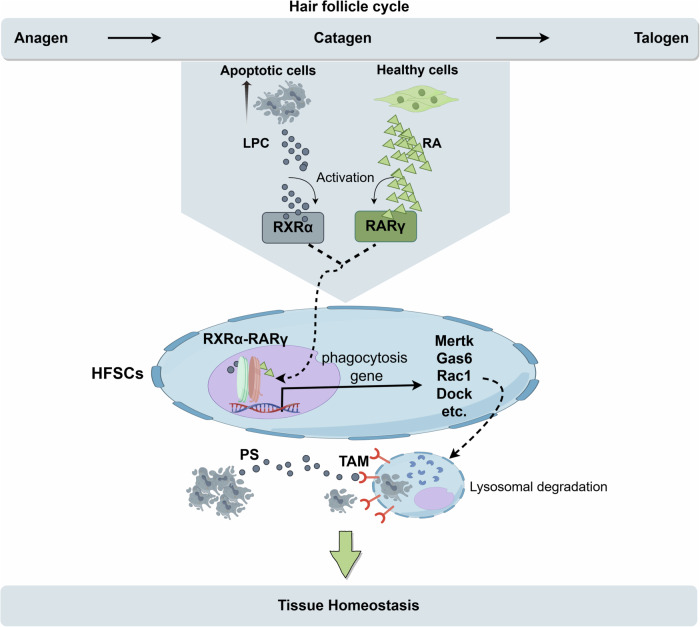
Mechanism of dead cell clearance by hair follicle stem cells (HFSCs) to preserve tissue health. Hair follicles go through the growth phase (Anagen), the regression phase (Catagen), and the resting phase (Telogen). During Catagen, a large number of apoptotic cells are produced. During this period, HFSCs show phagocytic ability and remove neighboring apoptotic cells to maintain normal structure and function of hair follicles. HFSCs show the highest phagocytic activity in the middle and late stages of the regression phase (CatVI-VII), and then gradually return to a resting state to play a regenerative function in the next hair follicle cycle. The phagocytic activity of HFSCs is regulated by the RXRα (retinoid X receptor α)-RARγ (retinoic acid receptor γ) complex. The activation of this complex requires two ligands: retinoic acid (RA) from healthy neighboring cells and lipid molecules released by apoptotic cells (such as LPC, a cleavage product of phosphatidylcholine). Under the joint action of RA and LPC, RXRα and RARγ form an active complex, translocate to the cell nucleus, and initiate the expression of a series of phagocytosis-related genes. Activation of the RXRα–RARγ complex leads to upregulation of TAM (TYRO3/AXL/MERTK) receptor expression. TAM receptors recognize the “eat me” signals (such as phosphatidylserine PS) on the surface of apoptotic cells. This recognition process initiates the phagocytic behavior of HFSCs, forming a phagocytic cup to encapsulate apoptotic cells, which then fuse with lysosomes and degrade apoptotic cells through lysosomal enzymes, avoiding secondary necrosis and maintaining tissue health. The figure was created with Figdraw.com

The study underscored RXRα as a pivotal mediator in synchronizing HFSCs phagocytic responses with their regenerative duties. Chromatin profiling indicated that RXRα dynamically regulates accessibility at phagocytic gene loci in response to LPC and retinoic acid. Moreover, through loss-of-function experiments targeting RXRα specifically in HFSCs, Stewart and colleagues observed that RXRα deficiency led to inefficient apoptotic clearance, resulting in apoptotic corpses left in the follicle, promoting secondary necrosis and potential inflammatory cascades. Thus, the RXRα–RARγ axis serves as a finely tuned switch that facilitates the rapid onset and offset of phagocytic activity, optimizing tissue clearance during catagen and preserving HFSCs’ capacity to re-enter quiescence in preparation for subsequent cycles.

In addition, it demonstrated that RXRα and RARγ activation by LPC and retinoic acid aligns HFSC phagocytosis with regenerative needs. RXRα ligands, such as arachidonic acid, in conjunction with retinoids, are shown to enhance TAM (TYRO3/AXL/MERTK) receptor expression, bolstering the HFSCs’ ability to recognize and engulf apoptotic cells. Intriguingly, RXRα–RARγ signaling appears contingent upon close contact with apoptotic cells, supporting a localized, self-limiting phagocytic model distinct from professional phagocytes that maintain continuous clearance in inflammatory or degenerative environments.^4^

The modulation of phagocytosis by RXRα has significant implications for tissue maintenance in immune-privileged environments. By balancing phagocytic duties with regenerative roles, RXRα–RARγ signaling in HFSCs may represent a targetable pathway for managing localized cell death in tissues with limited access to professional phagocytes. In contexts of chronic inflammation, degenerative disease, or impaired professional phagocyte function, pharmacologically modulating RXRα activity could theoretically enhance local apoptotic clearance while minimizing inflammation. RXRα and RARγ exist also in other types of stem cells, but their roles for dead cell clearance and tissue fitness have not been reported. Future studies targeting RXRα signaling in other non-professional phagocytes across tissue types could elucidate broader applications for RXRα in regenerative medicine.

Some cardiovascular diseases, like coronary heart disease, are often accompanied by apoptosis of cells. If stem cells can remove the apoptotic cells under appropriate conditions, they may slow down the disease progression, which may help understanding the pathogenesis and improving therapy of diseases, and will expand the stem cell research and application field in future.

This study provides valuable insights but also has some limitations. First, the study focused on a brief window in the hair follicle growth cycle, leaving the long-term impacts of repeated apoptotic cell clearance on HFSCs homeostasis unexplored. Future studies should examine the cumulative effects of apoptotic clearance on stem cell function and tissue health over extended periods. Additionally, while RXRα and RARγ play central roles, the study does not thoroughly investigate the mutual regulation and feedback mechanisms within these signaling pathways; for instance, potential variations in RXRα interactions with other nuclear receptors across tissues remain unclear. Finally, potential signaling interactions between professional and non-professional phagocytes, which may affect phagocytic efficiency and tissue adaptation,^5^ were not addressed. Clarifying these dynamics could reveal whether HFSCs phagocytic activity complements or operates independently of conventional immune responses.

In conclusion, this study unveils an unexpected role for HFSCs and novel molecular mechanisms in apoptotic cell clearance, enhancing our understanding of stem cell versatility in non-professional phagocytosis. Future research could explore the applicability and deeper validation of stem cell phagocytosis across diverse tissues, to provide a more comprehensive understanding of how stem cells maintain tissue health and open pathways to harness their phagocytic capacity in clinical settings.

## References

[1] Stewart, K. S. et al. Stem cells tightly regulate dead cell clearance to maintain tissue fitness. *Nature* **633**, 407–416 (2024). 39169186 10.1038/s41586-024-07855-6 PMC11390485

[2] Sukka, S. R. et al. Efferocytosis drives a tryptophan metabolism pathway in macrophages to promote tissue resolution. *Nat. Metab.* **6**, 1736–1755 (2024). 39242914 10.1038/s42255-024-01115-7 PMC11734744

[3] Raymond, M. H. et al. Live cell tracking of macrophage efferocytosis during Drosophila embryo development in vivo. *Science* **375**, 1182–1187 (2022). 35271315 10.1126/science.abl4430 PMC7612538

[4] Cummings, R. J. et al. Different tissue phagocytes sample apoptotic cells to direct distinct homeostasis programs. *Nature* **539**, 565–569 (2016). 27828940 10.1038/nature20138 PMC5807003

[5] Yuan, S. et al. Engineering Efferocytosis-Mimicking Nanovesicles to Regulate Joint Anti-Inflammation and Peripheral Immunosuppression for Rheumatoid Arthritis Therapy. *Adv. Sci.* **11**, e2404198 (2024). 10.1002/advs.202404198 PMC11267389 38810118

